# From QTL to candidate gene: Genetical genomics of simple and complex traits in potato using a pooling strategy

**DOI:** 10.1186/1471-2164-11-158

**Published:** 2010-03-08

**Authors:** Bjorn Kloosterman, Marian Oortwijn, Jan uitdeWilligen, Twan America, Ric de Vos, Richard GF Visser, Christian WB Bachem

**Affiliations:** 1 Wageningen UR Plant Breeding, Wageningen University and Research Centre, PO Box 386, 6700 AJ Wageningen, the Netherlands; 2 Plant Research International, PO Box 16, 6700 AA Wageningen, the Netherlands; 3 Centre for BioSystems Genomics, PO Box 98, 6700 AA, Wageningen, The Netherlands

## Abstract

**Background:**

Utilization of the natural genetic variation in traditional breeding programs remains a major challenge in crop plants. The identification of candidate genes underlying, or associated with, phenotypic trait QTLs is desired for effective marker assisted breeding. With the advent of high throughput -omics technologies, screening of entire populations for association of gene expression with targeted traits is becoming feasible but remains costly. Here we present the identification of novel candidate genes for different potato tuber quality traits by employing a pooling approach reducing the number of hybridizations needed. Extreme genotypes for a quantitative trait are collected and the RNA from contrasting bulks is then profiled with the aim of finding differentially expressed genes.

**Results:**

We have successfully implemented the pooling strategy for potato quality traits and identified candidate genes associated with potato tuber flesh color and tuber cooking type. Elevated expression level of a dominant allele of the β-carotene hydroxylase (*bch*) gene was associated with yellow flesh color through mapping of the gene under a major QTL for flesh color on chromosome 3. For a second trait, a candidate gene with homology to a tyrosine-lysine rich protein (TLRP) was identified based on allele specificity of the probe on the microarray. TLRP was mapped on chromosome 9 in close proximity to a QTL for potato cooking type strengthening its significance as a candidate gene. Furthermore, we have performed a profiling experiment targeting a polygenic trait, by pooling individual genotypes based both on phenotypic and marker data, allowing the identification of candidate genes associated with the two different linkage groups.

**Conclusions:**

A pooling approach for RNA-profiling with the aim of identifying novel candidate genes associated with tuber quality traits was successfully implemented. The identified candidate genes for tuber flesh color (*bch*) and cooking type (*tlrp*) can provide useful markers for breeding schemes in the future. Strengths and limitations of the approach are discussed.

## Background

The natural occurring genetic and phenotypic variation in plant genotypes of crop plants is at the core of today's breeding strategies. The ongoing effort to improve food quality has resulted in the mapping of many quantitative trait loci (QTLs) using traditional genetic marker technology. In contrast, the identification of the responsible gene(s) and their allelic variation and modes of action underlying phenotypic trait variation has proven difficult often due to the lack of understanding of the pathways involved or the complexity of the trait itself (i.e. polygenic traits). For commercial plant breeders the latter seems often of lesser concern as the availability of high quality genetic markers that can be screened in various populations is by and large sufficient. In potato breeding, there is a long list of desired traits and research interests that include plant growth and yield characteristics, disease resistance, tuber uniformity, size and shape, tuber content, nutritional value and post harvest tuber characteristics [1]. Although for many of these traits, major and minor QTLs have been identified in individual populations, the associated genetic markers identified are not necessarily useful in breeding schemes due to lack of sufficient resolution. Furthermore, genetic markers generated in one population can be quite distant from the physical location of the responsible polymorphism(s) in another and often difficult to translate to actual breeding material as the screened population does not always represent a similar genetic origin. Therefore, the clarification of the 'true' polymorphism(s) underlying trait variation is crucial if we want to understand and utilize the different evolutionary adaptation strategies that plants have taken which has provided us with the wealth of phenotypic variation observed today. The identification of the responsible gene underlying a trait QTL can lead to additional levels of information through subsequent allele mining or haplotyping across a range of cultivars.

Different approaches can be taken to find the genes explaining the observed QTL. Traditionally, positional cloning through fine mapping reduces the number of candidate genes that need to be tested in complementation studies. Similarly, *a priori *knowledge of the biochemical and signaling pathways involved can provide a short list of key regulatory and functional genes to be targeted for mapping and tested for association with the trait [2-4]. For many traits however, there is little knowledge on the associated pathways or the key regulatory steps and thus it remains difficult to identify a candidate gene directly linked to an underlying causative polymorphism.

The use of microarray technology for accurately scoring of differential gene expression within large populations has greatly enhanced the number of potential genes that can be screened and tested for association with a specific trait of interest [5-8]. Differential gene expression within a population can be considered as a quantitative trait that can result in the mapping of gene expression as a QTL or so-called eQTL [7]. Similarly, metabolite levels or protein levels can potentially be mapped as quantitave traits (mQTL's and pQTL's, respectively) [9]. Large scale expression profiling studies performed on plants (Arabidopsis, Barley, Wheat) has shown the potential of the methodology based on the large number of eQTL's and co-regulatory pathways that can be identified leading to network construction [10-13].

Gene expression variance can either derive from a polymorphism located physically near the gene (cis-eQTL) or indirectly from a distant location on the genome (trans-eQTL). Interestingly, cis-eQTL's appear to have a larger phenotypic effect than trans-QTLs [10,14]. The combination of genomic profiling and genetics has been referred to as 'quantitative genomics' or 'genetical genomics', and is expected to greatly advance our capabilities to resolve metabolic, regulatory and developmental pathways [13,15]. Although profiling techniques are now widely available for most important crop plants, screening of entire populations is still very expensive and not very cost-effective from a breeding point of view as the extent of phenotypic and genetic variation found for a particular quality trait is likely not to be captured in a single population, tissue type or time point. For the potato crop, high quality expression profiling platforms have now been established [16] and successfully implemented in screening for genetic diversity [17,18].

**Figure 1:**
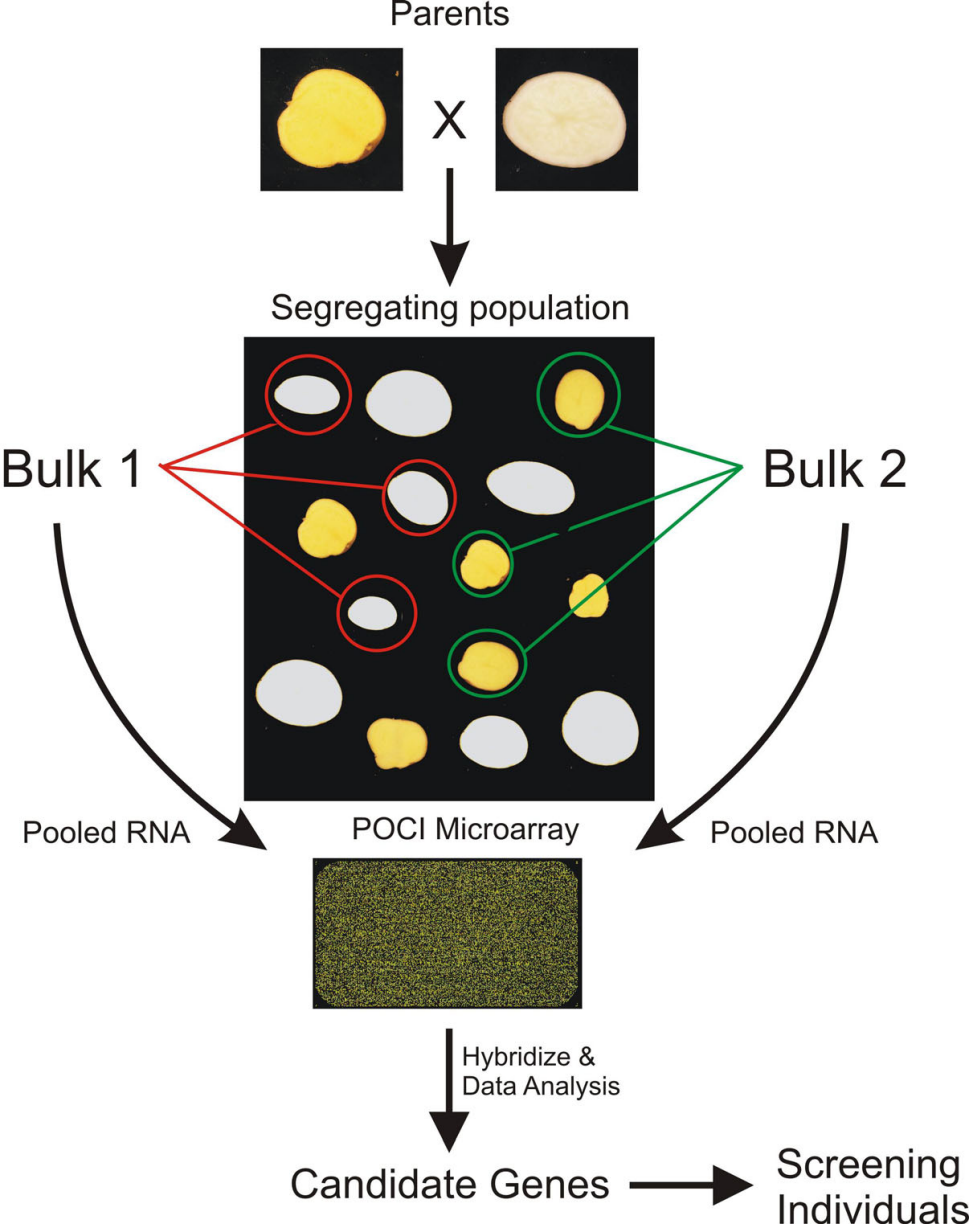
**Schematic overview of a typical BSA expression profiling experiment targeting tuber flesh color as an example**. Extreme individuals from a segregating population are identified and pools of RNA are collected. Gene expression is profiled for each of the pools using microarray technology. Genes displaying differential expression between the contrasting bulks are considered as candidate genes and further analyzed targeting the individual genotypes.

To reduce the number of hybridizations needed and thereby costs, pooling of RNA samples has been proposed or successfully implemented [15,19]. The utility of pooling has been assessed mainly from a statistical viewpoint and in general, pooling is thought to be efficient when pool sizes are sufficient, biological variability outweighs technical variation and independent samples contribute to multiple pools [20-23]. Here we present an approach that uses the power of quantitative genomics in revealing the most promising candidate genes for simple potato quality traits based on expression variation by implementing a pooling strategy. By profiling RNA pools, consisting of genotypes based on contrasting phenotypic or marker data, differentially expressed genes can be identified through association with a targeted trait. A schematic overview of this approach is shown in figure 1, representing a cross between two potato clones segregating for tuber flesh color. In this population, tuber flesh color ranges from white to dark yellow, and is in general attributed to the levels and classes of carotenoids [17,24]. Within any segregating population which has been properly phenotyped for a trait of interest, extreme individuals can be selected for pooling. Depending on the profiling technology used, harvested material (RNA, metabolite or protein extractions) can than be combined in equivalent amounts and analyzed using the appropriate platform. Ideally, multiple independent bulks should be formed to reduce the number of false positives. In order to find novel candidate genes underlying trait variation we performed 'pooled' gene expression profiling on three potato tuber quality traits; tuber flesh color, texture after cooking and free methionine content. The obtained expression data resulted in the identification of a large number of candidate genes that in some instances could be functionally linked to the phenotypic trait studied.

In several studies, the presence of a major tuber flesh color QTL on chromosome 3 has been reported. Two genes involved in the carotenoid pathway (*phytoene synthase*, *β-carotene hydroxylase*) have been associated with a potential role in controlling yellow flesh color and labeled as the candidate genes for the Y-locus [24-27]. For single QTL traits, like tuber flesh color, the selection of genotypes for each of the bulks can be based on the obtained phenotypic data. Here, extreme individuals based on flesh color are pooled together to get the largest possible phenotypic contrast. A similar pooling strategy can be envisaged for finding QTL associated metabolites and this was tested by measurement of carotenoid content of the same pooled material as used for RNA profiling.

A second trait that was analyzed using the pooling approach is texture after cooking. Texture of cooked potatoes is an economically important quality aspect and is generally characterized between the differences in mealy and non-mealy/waxy tubers. Textural changes occurring during cooking are mainly associated with cell wall and middle lamella structural components and the gelatinization characteristics of starch [28,29]. A mealy tuber is one which, while it retains its form on cooking, may readily be broken down to give a dry crumbly mash through separation of individual cells [30]. Genes labelled as candidate genes for tuber cooking type are usually cell wall biosynthesis or modification proteins [17]. However, it is unclear if the observed trait segregation can be attributed to these genes as there is little supporting evidence linking it to genetic variation.

When confronted with a polygenic trait, showing multiple QTLs for a particular trait, a more advanced pooling strategy is desired as more than one polymorphism or locus contributes to the observed phenotype in an independent manner. A more complex pooling strategy based on marker information and phenotypic data is tested for free methionine content in potato tubers which shows the presence of two QTLs on two different linkage groups. Methionine is one of the sulfur-containing amino acids and is the precursor of many essential bio-molecules [31]. Several attempts have been made to boost methionine content in potato tubers through increased biosynthesis or accumulation of methionine rich storage proteins with mixed results [32,33].

In this paper we present transcriptomics and metabolite data associated to several important potato tuber quality traits, segregating in a single diploid potato population. Lists of promising candidate genes were obtained using a pooling strategy and selected candidate genes were tested for association with the trait and a further subset was positioned on the genetic map and has led to some new insights into the regulatory pathways involved. Results are presented in terms of the quality of the candidate genes found, the validity of the pooling approach in potato, its limitations and potential pitfalls are discussed.

## Results

### Pooling single QTL traits - Tuber flesh Color

The accumulation of the different carotenoids is thought to be one of the major components to cause the distinctive yellow flesh color in potato tubers. Within the diploid C × E crossing population, potato tuber flesh color was quantitatively scored on a scale from 1 (white) to 9 (dark yellow/orange) as described in methods. The observed variance in flesh color found in our population can be largely explained (51.9%) by a single major QTL on chromosome 3 (LOD score 8.3). To test the pooling strategy for the identification of promising candidate genes underlying this QTL, we constructed RNA bulks consisting of extreme flesh color phenotypes (Figure 1). A total of four bulks were constructed, each consisting of 10 non-duplicated genotypes; two bulks for yellow fleshed tubers (Y1, Y2) and two bulks for white fleshed tubers (W1, W2). RNA pools were labeled and hybridized on the array as described in the methods section. The log2 ratios of yellow vs. white flesh bulks were calculated after normalization and passing significance cut-off levels. A consequence of using a pooling strategy is a reduction of the statistical power to perform reliable significance tests due to the low number of hybridizations performed. However, as we have four independent pools of RNA each representing 10 different genotypes, expression levels reflect the average of each pool in which expression outliers would have reduced effect. Therefore, consistent differentially expressed genes (>2-fold) across the four bulk comparisons, representing a total of 40 genotypes, were considered as candidate genes (see Additional file 1). We found 83 features/genes that were on average higher expressed in both yellow tuber bulks and 101 features exhibited lower expression in comparison to the white fleshed tuber bulks. The list of differentially expressed genes was screened by looking at the assigned putative gene functions that are based on sequence similarity. Most strikingly, a gene with high homology to *beta-carotene hydroxylase *(*bch*) exhibited strong differential expression and was, on average, more than 140-fold higher expressed in the yellow fleshed tuber bulks. As mentioned in the introduction, the *bch *gene has been associated with controlling tuber flesh color and was thus, based on the expression difference, a prime candidate for controlling flesh color in our C × E population and became the subject for further research.

**Figure 2:**
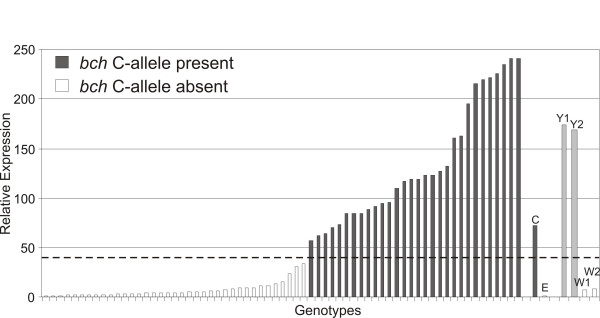
**Relative expression levels of the beta-carotene hydroxylase (*bch*) candidate gene associated with tuber flesh color**. Expression of the parental clones C and E, the yellow and white fleshed tuber bulks (Y1, Y2 - W1, W2) are indicated next to the set of individual genotypes from the C × E population. Genotypes containing the dominant *bch *B-allele are indicated in grey while genotypes lacking this allele are represented with white bars.

**Figure 3:**
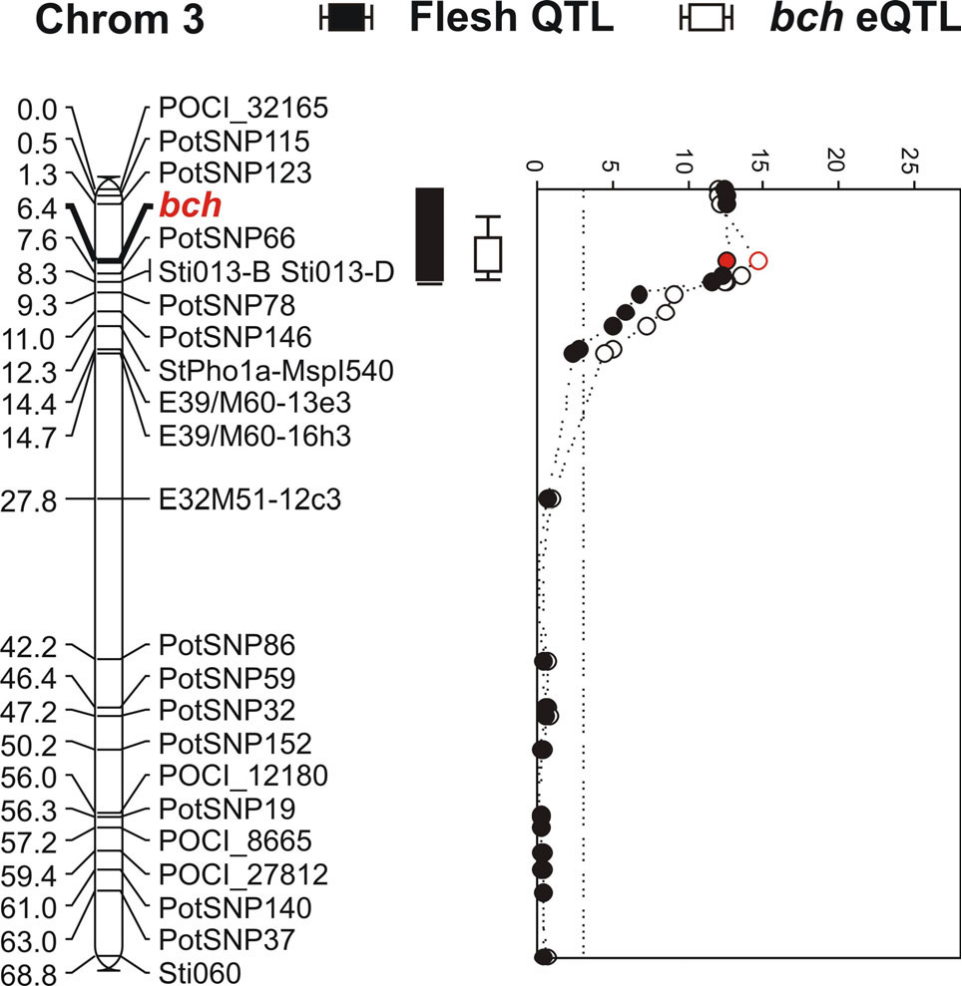
**QTL analysis of potato tuber flesh color scores and map position of the candidate gene *bch***. A major QTL for tuber flesh color was found on chromosome 3 with strong correlation to the *bch *gene marker (red). Variation in expression of the *bch *gene is explained by the presence of a QTL on the same genetic position as the gene itself (cis-eQTL).

The next step to validate any promising candidate gene that comes out of a pooling experiment is to confirm differential expression levels in the original RNA pools and the parental clones in a gene specific manner (qRT-PCR). If expression differences are confirmed, expression data for the individual genotypes should be obtained to check association levels of the candidate gene with the trait throughout the entire population. In the case of flesh color, gene specific primers for *bch *were designed and qRT-PCR was performed for the bulks, parental clones and finally the individual genotypes present in the population (Figure 2). The strong differential expression of *bch *between the yellow (Y1, Y2) and white (W1, W2) fleshed tuber bulks was confirmed. The variation in expression levels between the genotypes could clearly be separated into two categories; high and low expression levels with almost a 1:1 segregation pattern. We found a strong association of *bch *expression levels and yellow flesh color (r^2 ^= 0.6). As the C-parent exhibits high and the E-parent low *bch *expression, a genetic model arises were a dominant allele of the C-parent contributes to the determination of the final flesh color. Strikingly, all 20 genotypes present in the two yellow tuber bulks contain the dominant C-allele while for the white fleshed genotypes all 20 genotypes lack the same allele (Figure 2). Brown and co-workers [25] identified a dominant allele (B) highly correlated with yellow fleshed cultivars, showing enhanced levels of carotenoid accumulation. Sequence and SNP analysis indicates that the same allele is present in the C × E population. To further test *bch *as the candidate gene associated with tuber flesh color in the C × E population, the gene was positioned on the available genetic map. *Bch *maps directly under the QTL for flesh color on chromosome 3, strengthening its significance as the main candidate gene for controlling tuber flesh color (Figure 3). The observed variance in expression found with qRT-PCR was treated as a quantitative trait and produces a strong eQTL in the same genomic region as the flesh color QTL (Figure 3). As a result, the observed differential expression of *bch *and its high correlation with tuber flesh color provides strong evidence that *bch *is the candidate gene underlying the tuber flesh color QTL and thus the traditional Y-locus in potato as proposed by others [25]. Although, the presence of the dominant *bch *allele (B) is required for conferring a yellow flesh color, a significant proportion of residual variation in flesh color is still present. The origin of the expression differences appear to come from the gene location itself as we found a strong cis-eQTL on the same position. The complete coding region of the *bch *gene was sequenced and the allelic variations were determined. Sequence comparisons of the different alleles of bch confirmed that the observed expression differences does not arise from allele specificity of the probe on the array or the *bch *gene specific RT-primers (data not shown).

### Metabolic analyses of tuber flesh color

One of our main research interests is the unraveling of potato tuber quality traits related to nutritional quality. As described above, the accumulation of carotenoids in the tuber contributes significantly to the yellowness of the tuber and thus to its potential nutritional value. There are many different carotenoids present within the plant kingdom and also within a single plant species different carotenoids can be present in varying levels. To identify the key carotenoids in our potato population, we extracted carotenoids from the same bulks of plant material generated for the gene expression analysis. The use of the four bulks consisting of the two non-overlapping repeats for both white (W1, W2) and yellow tuber bulks (Y1, Y2) should not only provide a chance to associated variance of individual carotenoids content with tuber flesh color but also tests the validity of using a pooling approach for metabolite analysis. We also included both parental clones to test or support any genetic model that might arise from the obtained data.

Only four different carotenoids could be reliably detected (> ~5 μg/100 g FW) with zeaxanthin being the predominant carotenoid in both yellow flesh tuber bulks (187 and 245 μg/100 g FW for bulk Y1 and Y2, respectively) (Table 1). Zeaxanthin was about 5 times higher in the yellow (Y1, Y2) bulks as compared to the white (W1, W2) bulks. A second carotenoid that was markedly higher (~5 fold) in the yellow tuber bulks could not be identified with standard compounds at this time, but since its absorbance spectrum was highly similar to violaxanthin and its retention time slightly higher than the violaxanthin standard, we assume that this compound is a violaxanthin-ester. Although violaxanthin itself was elevated in one of the yellow bulks (Y1), this finding could not be confirmed by the second yellow tuber bulk (Y2) in which violaxanthin could not be detected. The fourth carotenoid, lutein, was slightly higher in the white fleshed tuber bulks (52 and 53 μg/100 g FW for bulk W1 and W2, respectively) in comparison to the yellow tuber bulks (38 μg/100 g in both bulks). The latter finding is interesting as all four carotenoids are elevated within the C-parent in comparison to the E-parent, consistent with the more intense yellow color of this clone (C-parent flesh score = 5, E-parent flesh score = 2). Tuber flesh color in the C × E population behaves transgressive, as the extreme yellow tuber genotypes are more yellow than the C parental clone. This is specifically evident from the levels of zeaxanthin, which are about 10-fold higher in the yellow bulks in comparison to the C-parent. However, the same is true for the white fleshed tuber bulks and the E-parent: the level of zeaxanthin in the white bulks is about 20-fold higher than in the E parent. The other carotenoids have similar concentration ranges between both parental lines and the genotype bulks.

**Table 1:** Carotenoid concentrations (μg/100 g FW) of the white and yellow tuber bulks and parental clones

	*μg/100 g FW*			
	Lutein	Zeaxanthin	Violaxanthin	Violaxanthin-like
**White Bulk 1 (W1)**	52 ± 1^a^	31 ± <1	6 ± 1	10 ± <1
**White Bulk 2 (W2)**	53 ± 6	59 ± 10	7 ± <1	11 ± <1
**Yellow Bulk 1 (W1)**	38 ± <1	**187 ± 17**	14 ± 1	**60 ± 5**
**Yellow Bulk 2 (W2)**	38 ± 3	**245 ± 15**	Nd^b^	**36 ± 4**
				
**C parent**	62 ± 5	**19 ± 4**	19 ± 1	**110 ± 6**
**E parent**	29 ± 2	2 ± <1	8 ± 1	12 ± <1

^a ^Standard deviation is based on two technical repeats for the bulks and three repeats for the parental clones

^b ^Nd - Below detection limit

From this pooling experiment it became evident that both zeaxanthin and the unidentified violaxanthin-like carotenoid are the main contributors to the yellow flesh colors within this population. It remains unclear whether both carotenoids need to be present at high concentration in order to support a dark yellow color, as well as to what extend the accumulation of the individual carotenoids is genetically controlled. In light of the observed elevated level of *bch *gene expression in the yellow tuber bulks, these findings are consistent with a genetic model involving the dominant *bch *allele (B) responsible for the overall increase in carotenoids content downstream of β-carotene.

### Texture after cooking

Texture of cooked potatoes is an economically important quality aspect and is generally characterized as the differences in mealy and non-mealy/waxy tubers. The genetic components and genes involved in potato tuber cell wall characteristics in relation to the differences in cooking type have not been fully understood and there is a clear lack of high-quality candidate genes. Within the C × E population the degree of mealiness after cooking of the tubers was visually scored on scale from 1 (firm) to 6 (extreme mealy) for two harvest years (methods). Linkage analysis reveals the presence of a single QTL on the long arm of chromosome 9 for both harvest years (max. LOD score, 5.7 with ~25% explained variance). Based on these data sets, extreme genotypes were selected for pooling to perform bulk-segregant-analysis (BSA) expression profiling. Similar to the experimental setup as described for tuber flesh color, four independent bulks were generated representing both the mealy and firm tubers after cooking. Gene expression profiling of the four contrasting bulks was performed as described in the methods section. After data processing a total of 78 differentially expressed genes remained with at least a two-fold difference in the bulk contrasts. The complete list of differentially expressed genes associated with tuber mealiness after cooking is given in Additional file 2. A larger proportion of the differentially expressed genes (62 features) were more highly expressed in the firm tuber bulks in comparison to the mealy tuber bulks (16 features). Amongst the list of candidate genes, unigene contig Micro.187.C2 http://pgrc.ipk-gatersleben.de/poci showed high sequence homology to a class of tyrosine and lysine rich cell wall proteins (TLRP) previously identified in tobacco and tomato and was on average more than 5-fold higher expressed in the firm tuber bulks. This class of cell wall proteins is characterized by high level of tyrosine and lysine residues and contains a highly conserved N-terminus signal peptide targeting the protein to the cell wall. Of interest is the fact that these type of proteins are thought to be involved in cross-linking other proteins to the cell wall making them insoluble [34]. Therefore the identified candidate gene, named StTLRP, can be tentatively linked to a role in influencing tuber firmness after cooking and was selected for further analysis.

**Figure 4:**
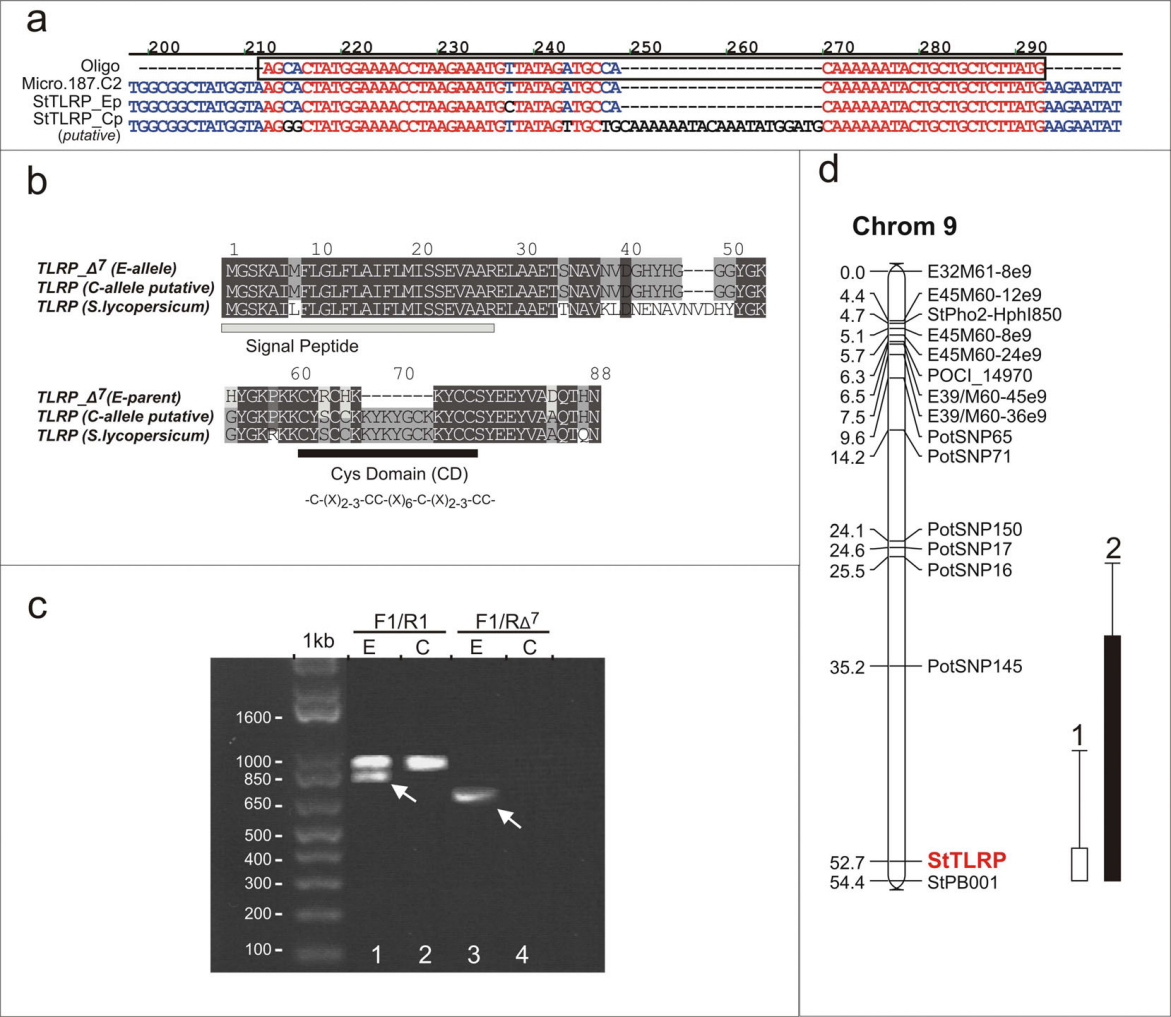
**Sequence analysis and map position of candidate gene *StTLRP *associated with potato tuber cooking type**. Alignment of cDNA sequences representing the identified allelic variation of the TLRP gene and the oligo sequence (micro.187.c2) present on the microarray (a). Alignment of the predicted protein sequences of the potato TLRP allele variants and the tomato homolog (CAA54561). The signal peptide and the Cys-binding domain are indicated (b). Marker development based on the variation in length of PCR products revealing the allelic variation found between both parental clones and the presence of deletion site in the unique E-allele (TLRP_Δ7) (indicated with arrow lane 1). Allele-specific amplification of the TLRP_Δ7 allele in the E-parent (lane 3) (c). Map position of the potato TLRP gene on the long arm of chromosome 9 under the QTL for cooking type analyzed for two years (1, 2) (d).

Initially, the same protocol was followed as was done for tuber flesh color and gene specific primers were designed followed by qRT-PCR on the bulks, parents and individual genotypes. Surprisingly, amplification of the targeted gene region was only detected in a small number of genotypes indicating the possibility of allele specific amplification. As it turns out, the unigene sequence on which the oligo probe was designed represents an allelic variant of one of the potato *TLRP *genes present in the EST databases. Sequence analysis led to the discovery of a 21 bp deletion in the coding region unique for the paternal E-allele within the region of the oligo design (Figure 4a). Only the genotypes containing this allele are able to hybridize to the oligo and contribute to the overall hybridization signal of the bulks. We refer to this allele as TLRP_Δ7 after its 7 amino acid deletion within the coding region (Figure 4c). Within the genotypes that made up the extreme mealy tuber bulks, only 3 out of a total of 20 (2 × 10) genotypes contained this allele while 15 out of 20 genotypes from the firm tuber bulks contained the allelic variant which explains the observed differential expression pattern found between the four bulks. Thus, the presence of the allelic *TLRP *variant is negatively associated with the degree of tuber mealiness. This correlation is not as strong (r^2 ^= -0.45) as found for the candidate gene linked to tuber flesh color (*bch r*^2 ^= 0.6).

We then went on to identify the remaining alleles present in our backcross population in order to be able to test their contribution and dosage within the individual genotypes. However, the StTLRP gene is part of a highly homozygous gene family impeding additional allele identification. Due to the high number of different haplotypes that could be identified within the TLRP gene family we were unable to specify the complementing alleles. None of the obtained sequences however, contained the same deletion site and overall homology showed strong conservation on the nucleotide and amino acid level. Therefore, the gene sequence deriving form the C-parent is labeled as a putative allele (Figure 4a, b). Genomic sequencing of the intronic regions revealed the presence of an additional deletion site of around 115 bp occurring only in the identified TLRP_Δ7-allele. Based on these deletions it was relatively easy to develop discriminating PCR markers for allele scoring within the population in order to map StTLRP onto the genetic map (Figure 4c). *StTLRP*_Δ7 was positioned on the long arm of chromosome 9, directly under the QTL for potato tuber cooking type (Figure 4d). It is still uncertain to what extent the identified allele is responsible in determining potato tuber cooking type as it only explains ~25% of the observed variance in the population and inclusion of the candidate gene as an additional genetic marker does not increase the overall LOD scores. However, the identified 21 bp deletion is within the putative CD-domain region thought to be crucial for cross-linking of proteins within the cell wall [34]. Based on its putative function and map position *StTLRP *remains a strong candidate gene for the QTL on chromosome 9, and functions as good example how allele hybridization specificity can lead to novel candidate genes associated with a genomic region of interest.

### Pooling polygenic traits - Methionine content

As shown for the 'flesh color' and 'texture after cooking' QTLs, the construction of expression pools for simple traits can be solely based on the available phenotypic data and is relatively straightforward. The majority of important quality traits in crop species however, are usually more complex and often polygenic, and when pooling the individual genotypes for BSA profiling, any such information should be taken into account where possible.

**Figure 5:**
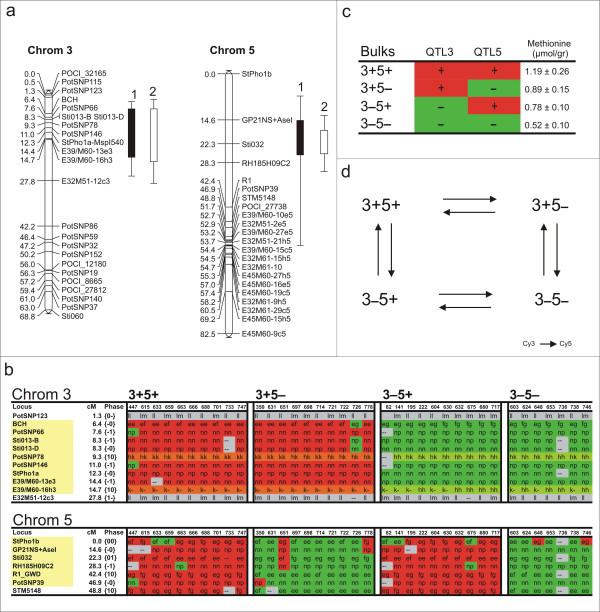
**BSA expression profiling of methionine content in tubers**. QTL analysis of tuber methionine content for two consecutive years (year1, 2) in the C × E population showing significant QTLs on linkage groups 3 and 5 (a). Graphical genotyping of the markers scores in the genomic region representing the methionine QTL on linkage group 3 and 5. Four bulks were constructed (3+5+, 3+5-, 3-5+, 3-5-) that represent either the presence or absence of the identified QTLs (b). Average methionine content of the four bulks was calculated based the genotypes scores (c). Hybridization scheme implementing a loop design for the four constructed bulks (d).

To test the pooling approach for a polygenic trait we targeted tuber methionine levels in stored tubers. Methionine content was determined within the C × E population over two years (Methods). Two significant QTLs were detected for both years on chromosome 3 and 5 (Figure 5a). In such a case, besides the phenotypic data also the marker data spanning the different QTL regions is taken into account during the bulk design (Figure 5b, c). Here again, four bulks are constructed but now representing either the presence (+) or absence (-) of both QTLs (3+5+, 3-5-), or the presence of a single QTL, either QTL3 (3+5-) or QTL5 (3-5+) based on the available marker scores (Figure 5b). Due to the low number of markers on the genetic map on chromosome 5 and the fact that the QTL lies within close proximity of the centromeric region (Tang, unpublished data), the methionine QTL spans a relative large region (47 cM) while the QTL on chromosome 3 is limited to a genetic region of only 8.3 cM. In terms of physical distance, and thus the number of targeted genes in these regions, there is currently little information available. The marker scores for the individual genotypes are color coded and indicate the presence or absence of recombination events across the significant QTL regions. Due to our small population size, genotypes nr 651, 726 and 778 were included in the bulk although some recombination events within the targeted region appear to have occurred. The predicted averaged methionine content based on the individual genotype data are listed and reflect either high, intermediate or low methionine content (Figure 5c). Hybridizations were performed using a loop design to allow testing for interaction of genes with either one or both of the QTLs (Figure 5d). A biological meaningful association was considered when there was at least a two-fold difference in expression levels (p < 0.05) between the 'presence' and 'absence' of the individual QTLs in the four bulks. A total of 32 'genes' were significantly associated with the QTL on chromosome 3 (20 negative and 12 positive) while a total of 100 'genes' exhibited significant association (58 negative and 42 positive) with the methionine QTL located on chromosome 5. All significantly associated genes are presented in Additional file 3.

Considering our current knowledge on methionine biosynthesis and downstream routes in plants, the list of candidate genes was screened but revealed few obvious candidate genes. Sequencing of the potato genome is well underway and in particular chromosome 5 has been the focus of several sequencing efforts. Estimates of the total coverage of chromosome 5 with sequence data is currently around 60-80% and it is thus warranted to perform a blast search of our candidate genes against the available sequence data. We found that 31 out of the 100 differentially expressed genes had a positive hit with one or more BAC sequences located on chromosome 5 with a sequence homology greater than 90% and e-value < 1.0E^-50 ^masking for repetitive sequences. BAC clone names associated with chromosome 5 are indicated in Additional file 3. In general we found more hits with the positive associated genes on chromosome 5 in comparison to the negative associated candidate genes. The significance of the remaining candidate genes for which no BAC hit was found remains uncertain and potential explanations will be addressed in the discussion.

**Figure 6:**
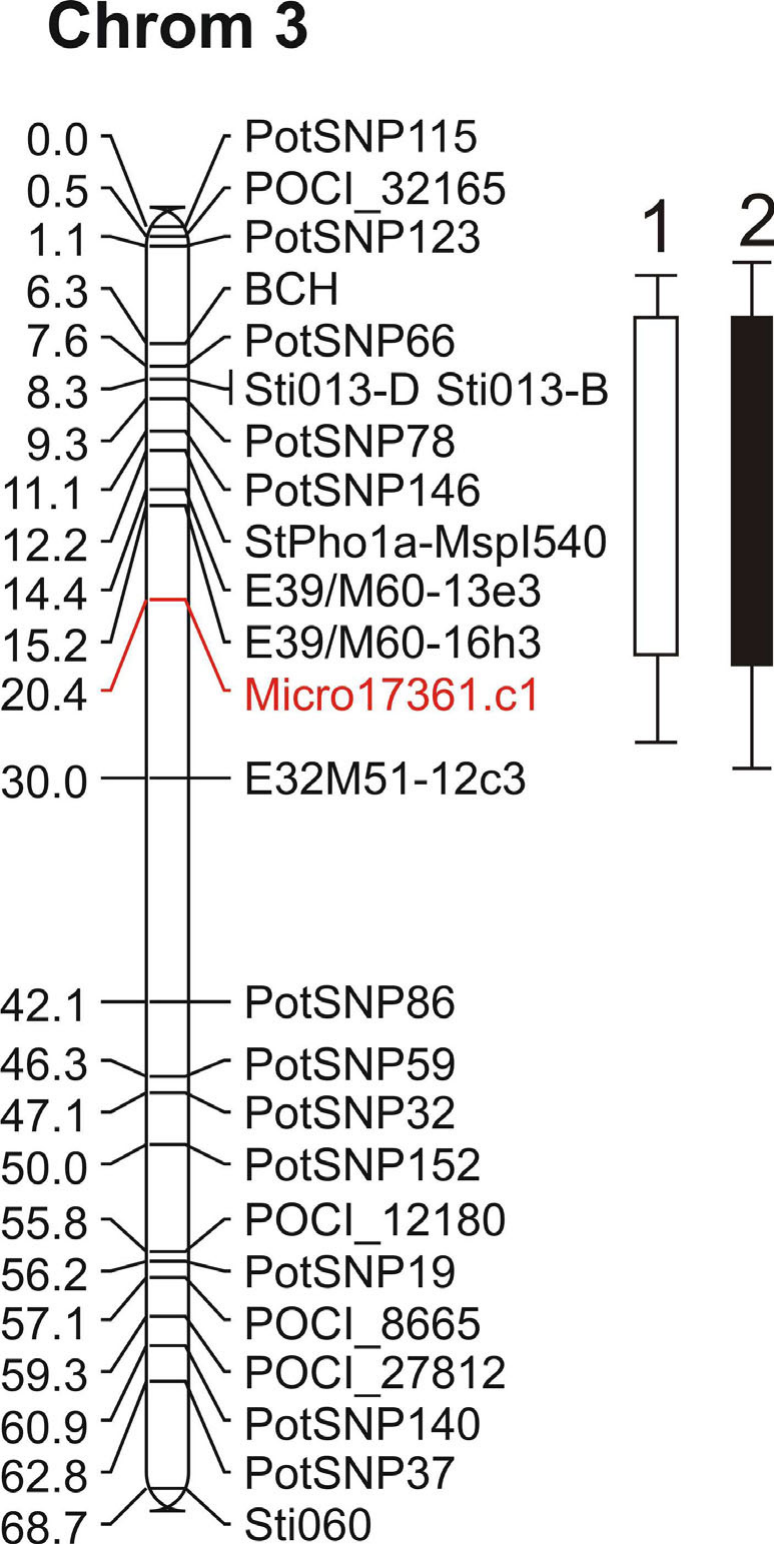
**Mapping of a candidate gene for methionine content**. Map position of candidate gene *micro.17361.c1 *on linkage group 3, indicated in red, and the identified QTL for methionine content for two years spanning the equivalent genomic region.

Unlike chromosome 5, sequencing efforts of chromosome 3 have been minimal up to now and therefore the lack of BAC hits with any of the candidate gene sequences is not surprising. To show that the pooling strategy based on genetic marker information works for both targeted genomic regions (chromosome 3 and 5), we designed primers for a small subset of genes uniquely associated with chromosome 3. From the initial tested set of primers, we could confirm the differential expression pattern of unigene *micro.17361.C1 *in the different bulks and this gene was subsequently selected for screening on the individuals. Interestingly, for a subset of the genotypes, expression levels did not rise above the detection limits whilst the majority of the genotypes expression showed stable expression. In the RT-PCR, absence of expression concerns only those genotypes present in the low methionine tuber bulks associated with the QTL on chromosome 3. As equal expression levels were detected in both the parental clones and by looking at the neighboring markers present on the genetic map, we can infer that the common allele (allele 1), present in both parents, is expressed. In the complete absence of the common allele within the F1 genotypes, no expression is detected. The genetic marker was subsequently placed on the genetic map as a bridge marker on chromosome three in close proximity of the identified QTL region for methionine content (Figure 6). The qRT-PCR data of the individual genotypes present in the bulks thus confirms the on average 'reduced' expression levels found for the low methionine bulks associated with QTL3 (see Additional file 3). It seems therefore likely that the oligo on the array has a similar specificity for allele 1. Additional PCR markers were generated that confirmed the segregation pattern as found for the qRT-PCR (data not shown) and subsequently allowed confident mapping of the candidate gene on the genetic map. The positioning of the candidate gene near the targeted QTL shows that through BSA expression profiling novel genes/markers can be associated with individual QTLs in case of a polygenic trait. The sequence of contig *micro.17361.c1 *displays no significant homology to any functional annotated protein present in the databases. A new QTL analysis of the methionine data using the extended genetic map now including the candidate gene marker does not increase the LOD scores. Identification and scoring of the remaining alleles for *micro.17361.c1 *should be established before a decision on the potential of this candidate gene can be made.

## Discussion

The location of a QTL on a genetic map highlights the genomic region where at least part of the observed phenotypic variation originates. Genetic variations underlying these QTLs such as duplications, indels or SNPs, can influence different aspects of cell biology by affecting gene transcription and protein activity on many different levels both directly and indirectly. Large scale transcriptomics analysis of segregating populations or recombinant inbred lines can reveal associations between gene expression and the quantitative trait data or QTLs but is relatively costly when screening whole populations over multiple years and different time points or tissue types [10-12,35]. By using a pooling approach we can rapidly screen a segregating potato population for the association of variance in gene expression with quantitative trait data. Through pooling one reduces the number of hybridizations needed and increases flexibility when additional time points or tissues are desired. The latter is crucial and also raises one of the first important questions to be answered before designing a pooling experiment. At what stage of the life cycle is the targeted phenotype being developed and should sampling thus ideally take place? For example, tuber shape is determined in the early stages of tuber organogenesis, while tuber cooking type is more likely to be controlled at later stages of tuber bulking and storage. Similarly, if one is interested in the degree of stolon branching or time of flowering, there is little use for expression profiling data based on tuber material. The proposed strategy by Li et al, [36] using a generalized genetical genomics approach, integrates multiple factors (environment, tissue type) into the experimental design but does not necessarily reduce the number of samples that need to be processed.

The expression profiling of the pools presented here was performed using harvested and stored tubers as we are primarily interested in post harvest tuber quality traits. Moreover, due to the strong segregation of plant maturity type in the C × E population, the use of younger tubers would introduce a strong bias, based on the variation in the physiological age of the tubers and thus gene expression. Therefore, the traits presented in this study were profiled using fully matured tubers derived from a field experiment so that they would resemble a similar physiological state. Despite these precautions we cannot predict to what extent the transcriptome is affected due to the shorter life cycle of a number of the genotypes used in this study.

Planning a BSA profiling experiment in populations for which a genetic map is available provides certain advantages as already heritability and QTL studies can reveal the presence of any genetic factor(s) associated with the trait. For single QTL traits the pooling procedure is straightforward as phenotypic data can be used to construct the bulks. Having the availability of a genetic map provides additional level of information as both phenotypic data and haplotype information can be used in the selection process. The number of candidate genes that are differentially expressed between contrasting pools varies and is dependent on the pool size, population structure and the trait targeted. The C × E population is a relatively 'wild cross' with highly heterozygous parental clones resulting in the segregation of many quantitative traits. The presence of high genetic variation levels is likely to have an impact on the number of differentially expressed genes in the RNA pools. The design of additional bulks or number of genotypes represented in the individual pools should reduce the number of false associations; however, this is not always possible due to the size or trait segregation pattern of the available populations. If we compare the three profiling experiments described in this paper we found only a single feature that showed strong association with all three traits and was considered a clear false positive. An additional 19 features/genes were found differentially expressed in more than one trait association. This number is surprisingly low considering that for two traits we are targeting the same linkage group (chromosome 3 for both flesh color and methionine content). The number of candidate genes differentially expressed and associated with the quantitative trait data is still considerable and makes candidate gene selection difficult. However, putative gene annotation can certainly aid in the discovery of promising candidate genes as was the case for tuber flesh color, where one of the candidates showed homology to *beta-carotene hydroxylase *(*bch*) thought to be the primary candidate gene for the traditional Y-locus on chromosome 3 [25,26]. The finding of the *bch *as a candidate gene may therefore not be entirely unexpected; completely novel however is the finding that the dominant *bch *allele (B) appears to act trough enhanced expression levels. The variation in expression levels of the *bch *gene is cis-acting as exemplified by the presence of a large eQTL on the genetic position of *bch *(Figure 3). An elevated expression level of *bch *gene is likely to result in an increase of total enzyme activity, driving the conversion of β-carotene to zeaxanthin although this needs to be confirmed with additional experiments. Given the fact that various oxygenated carotenoids, but not β-carotene itself, show elevated levels in the yellow tuber bulks (Table 1), BCH activity appears to enhance the overall flux through the carotenoid biosynthesis pathway. Measurements of the carotenoids content of the individual genotypes from the bulks should provide more insight into their variation as the mere presence or absence of the dominant B allele cannot fully explain the range of yellowness observed. Similarly, although the presence of the dominant B-allele seems to be required for conferring yellow tuber flesh, additional copies of this allele does not increase the yellow flesh color any further, indicating additional (genetic) factors are probably involved [25]. Cloning of the promoter regions of the various alleles should provide more insight into the genetic variation underlying this expression difference.

In contrast to the *bch *gene, the candidate gene identified for tuber cooking type (*StTLRP*) and methionine content (*micro.17361.c1*) were not based on true expression differences but rather hybridization specificity of the designed oligo (Figure 4a). In light of our primary goal which is candidate gene finding, this poses no problem as any association between the hybridization signal and a quantitative trait can be potentially interesting. However, from a strictly biological sense, any observed variation in gene expression levels between evolutionary distinct genotypes should be treated with care as was shown in other studies [37-39]. Even more so, as potato is a highly heterozygous crop and the number of SNP's in this species has been estimated to be as high as 1 SNP per 24 bp of genomic DNA (bi-allelic) [40]. Long oligo based array platforms such as Agilent's, are sensitive to mismatches between oligo and their labeled targets. A study by Hughes et al., [41] revealed that the position of the mismatch using this profiling platform can be crucial for its effect on hybridization efficiency. It is thus to be expected that the oligo design based on sequence information from a highly heterozygous crop like potato brings forth hybridization specificity issues due to the huge amount of allelic variation present. In the most extreme case this would lead to the design of an allele specific oligo as was identified for the potato TLRP gene. The allele specificity of oligo *micro.187.c2 *(TLRP) was confirmed by hybridizing genomic DNA of both parents to the array where data analysis revealed significant signal in the E-parent only (data not shown). From a gene discovery point of view the high degree of genetic variation in potato can be considered an advantage as not only gene expression variation but also difference in hybridization efficiency can lead to the discovery of novel associated candidate genes. On the other hand, the number of 'false' positives also increases as genetic variation present in genes that are located in close proximity of the targeted locus are more likely to also exhibit differential 'expression'. Again, with a pooling approach the size of the targeted genomic region depends on several issues that include; the quality of the phenotypic data obtained and marker density in the QTL region, pool size and the occurrence of sufficient recombination events within the selected genotypes.

A non-targeted approach for candidate gene finding in potato as described here provides the possibility to discover novel genes or associated pathways. However, given the large number of differentially expressed genes in any profiling experiment and the often poor annotation it also poses a problem as the quality of the candidate gene remains obscure and requires additional experiments.

In the methionine profiling experiment we mapped a candidate gene (micro.17361.c1) with unknown function on chromosome three in close proximity of the QTL for methionine content (Figure 6). Although in this case the addition of a new marker did not narrow the QTL region, addition of more marker information or candidate genes on to the genetic map could increase significance and prove useful in marker-assisted breeding. A great advantage in this respect will come from the completion of the potato genome sequence in the near future. Obtained candidate genes can than directly be located on the genome and cis- and trans-acting factors can be distinguished. A subset of the candidate genes associated with the methionine content on chromosome 5 could already be placed on sequence data anchored on an ultra high density map of potato [42]. In this manner strong candidate genes for a quality trait can more easily be identified, while genes located on other linkage groups can be either considered as false positives due to pleiotropic effects and/or act downstream of the targeted locus.

The ability to physically place differentially expressed genes on the targeted genomic region shows that the pooling strategy for a polygenic trait, including marker scores does allow the screening of independent linkage groups associated with the trait within a single experiment. In the case of methionine content, two QTL's were targeted using a four pool design which seems to be the limit with the current population size (94 individuals). Already within the bulks constructed for methionine content, genotypes were included that exhibited recombination events in the targeted area that may result in noise (Figure 5b).

In case of the availability of larger populations, one could envisage experimental designs targeting three or more QTLs within the same experiment although the number of hybridizations needed to obtain statistical significance would need to increase as well and pooling would no longer be beneficial. It is clear that performing a genome wide genetical genomics experiment provides the researcher with additional tools to study the data and perform multi-loci interactions and possibly network construction as shown by others [10]. Unraveling of complex traits would require such a broad approach and pooling alone would not suffice in identifying suitable candidate genes. Based on the relative large number of differentially expressed genes that come out of any pooling experiment, it remains difficult to classify these candidate genes as either true candidate genes, false positives or genes that are merely physically located near the targeted genomic region, without the additional analysis of the individual genotypes and allele mining. The true identification of any gene underlying a quality trait remains dependent on additional research requiring at least a basic understanding of gene function as was available for the candidate gene linked to tuber flesh color and cooking type.

BSA expression profiling is not limited to populations for which a genetic map is available and thus novel populations arising from breeding programs can be quickly screened for trait associated gene expression. Transcript profiling has not only the potential to identify a set of candidates associated with quality traits but can provide a direct link to the underlying biological mechanism. For example, in the case of potato tuber flesh color, the observed transcription regulation at the *bch *locus would have been missed if one only focuses on the allelic segregation pattern.

## Conclusion

The identification of candidate genes underlying trait variation remains a great challenge in modern plant breeding. Here we show the identification of novel candidate genes associated with quantitative potato tuber traits by pooling extreme individuals (BSA expression profiling) in a segregating population. A pooling approach provides a quick way of screening populations for candidate genes associated with quality traits in comparison to screening of individuals. We identified promising candidate genes for controlling potato tuber flesh color (*bch*) and cooking type (*tlrp*). In addition, we performed a profiling experiment on a polygenic trait (methionine content) by pooling individual genotypes both on phenotypic and marker data leading to the identification of associated candidate genes linked to the different linkage groups.

## Methods

### Plant material

A subset of the diploid backcross population (C × E) consisting of 94 individuals was used in the experiment derived from the original cross between C (USW533.7) and E (77.2102.37) as described elsewhere [43]. Clone C is a hybrid between *S.phureja *and *S.tuberosum *dihaploid USW42. Clone E is the result of a cross between Clone C and the *S.vernei*- *S.tuberosum *backcross clone VH^3^4211. All clones were grown in multi-year repeats in the field (Wageningen, The Netherlands) during the normal potato-growing season in the Netherlands (April-September). For each genotype, tubers were collected from three plants and were either used for phenotypic analysis or mechanically peeled and immediately frozen in liquid nitrogen before being ground into a fine powder and stored at -80°C.

### Phenotypic and metabolite analysis

Potato tuber flesh color was visually scored on a scale from 1 (white) to 9 (dark yellow/orange) in three repeats consisting of two plants each for one harvest year. Flesh scores were averaged over the three repeats. Textural changes of tubers after cooking were determined on two consecutive harvest years. Harvested tubers derived from field experiments with three replicates for each genotype, each consisting of two plants. Tubers of the three replicates were harvested and stored for three weeks in controlled conditions before being analyzed. Three tubers of each sample were peeled and steam-cooked for 20 minutes, after which the texture was visually scored on a scale ranging from 1 (firm/non-mealy) to 6 (extreme mealy).

Carotenoid profiles were determined for the four bulks and both parental lines in respectively two and three repeats. Carotenoids were extracted and analyzed by HPLC with photodiode array (PDA) detection, according to the protocol described in [44]. In short, 0.5 g FW of ground and frozen tuber material was extracted with methanol/chloroform/1 M NaCl in 50 mM Tris (pH 7.4) in a ratio of 2.5: 2: 2.5 (v:v:v) containing 0.1% butylated hydroxytoluene (BHT). After centrifugation, the samples were re-extracted with 1 ml chloroform (+ BHT). The chloroform fractions were combined, dried under a flow of N_2 _gas and taken up in ethyl acetate containing 0.1% BHT. Carotenoids present in the extracts were separated by HPLC using an YMC-Pack reverse-phase C30 column and analyzed by PDA detection with wavelength range set from 240 to 700 nm. Eluting compounds were identified based on their absorbance spectra and co-elution with commercially available authentic standards (neoxanthin, violaxanthin, antheraxanthin, lutein, zeaxanthin, β-cryptoxanthin, ε-carotene, α-carotene, β-carotene, ζ-carotene, δ-carotene, prolycopene and all-trans lycopene. Limit of detection was about 5 μg/100 g FW and technical variation (6 independent extractions and analyses of the same tuber powder) was less than 8%.

Methionine content of tubers was determined over the two years using the ground tuber material that was stored at -80°C as described above. For soluble amino acid extraction, 150 mg of the ground tuber material was weighed and 500 μl of 70 mM phosphate buffer (pH 7.0), containing 1 mM dithiothreitol (DTT) was added. Three μl of 2 mM norleucine were added to the extract as internal standard. The samples were mixed for 3 min after which 2.5 ml of a mixture of methanol, chloroform and water (12:3:5) was added. The samples were mixed again and 500 μl of deionised water was added followed by centrifugation for 25 min at 3000 × g. The water phase was transferred to a new tube and the extraction was repeated twice with 2.3 ml of water. All three water phases were combined and transferred to a new tube and freeze dried. The freeze dried material was dissolved in 1 ml of water and centrifuged at 12,000 × g for 30 min to remove any insoluble substances. Amino acid analysis was performed with a BioChrom 20 (Amersham Pharmacia Biotech). One hundred and fifty μl of 0.2 M lithium citrate buffer (pH 2.2) was added to 150 μl of the sample and 40 μl of the mixture was loaded onto the ion-exchange column (Ultrapac 8 resin lithium form, I = 200 mm, d = 4-6 mm). A stepwise elution by 5 lithium citrate buffers (pH 2.8, 3.0, 3.15, 3.5, 3.55) was employed and the amino acids were detected with ninhydrin reagent and the concentration expressed as mg g^_1 ^dry weight (DW).

### Microarray hybridizations and data processing

RNA was extracted of the 94 individuals using a hot phenol method described previously [45]. Equal amounts of total RNA from the individual genotypes was pooled to generated RNA bulks to be used in the expression profiling studies or downstream quantitative RT-PCR after purification and DNAaseI treatment using the RNeasy Minelute Spin columns (Qiagen). RNA pools generated for the flesh color and texture profiling experiments were labeled using the low RNA input linear

Amplification Kit, PLUS, Two color (Agilent technologies) according to the manufacturer's protocol starting with 2 μg of purified total RNA. RNA pools for the methionine expression profiling were labeled as described elsewhere [16]. Hybridization and washing was performed according to the Agilent's two color hybridization protocol with the following change: 1 μg of labeled cy5 and cy3 cRNA was used as input in the hybridization mixture. Slides were scanned on the Agilent DNA Microarray Scanner and data extracted using the feature extraction software package (v9.1.3.1) using a standard two-color protocol. Methionine bulks profiling was done on the 1 × 44 k slide format, while the profiling for flesh color and texture bulks used the 4 × 44 k format with extended dynamic range (XDR) scanning option. ^2^log Cy5/Cy3 ratios were calculated after passing quality check and minimal expression levels. The flesh color and texture bulks comparisons consist of two technical replicates (swop dye) and a biological repeat (two independent bulk comparisons). Differential expression of genes was considered when both technical and biological repeats showed consistent differential expression greater than 2-fold. The ^2^log ratios for the eight methionine hybridizations were imported into Genstat^® ^11.1 for statistical analysis (ANOVA) and calculation of expression estimates and standard errors for each of the four bulks. Only significant associations of gene expression (greater than 2-fold, p < 0.05) with either the QTL on chromosome 3 or chromosome 5 were considered as candidate genes. All expression data has been deposited in ArrayExpress (E-MEXP-2443, E-MEXP-2441).

### Quantitative RT-PCR

Gene specific primers for candidate genes were designed using the beacon designer™. cDNA synthesis of genotypes and RNA bulks was carried out using iScript One-Step RT-PCR kit (Bio-Rad). qRT-PCR using SYBR green was carried out in duplicates on the iCycler (Bio-Rad) and quantified with the Bio-Rad iQ5 optical system software. As a reference a eukaryotic translation initiation factor 3E-like gene from potato was used. All primer sequences are listed in Additional file 4.

### QTL and candidate gene mapping

Based on an earlier version of the C × E genetic map [46], a simplified map was made using mapping software Joinmap 4.0^® ^[47], based on 94 individuals with few additional markers (Kumari, unpublished results). Genetic markers for candidate genes targeted for mapping were either PCR-based (*StTLRP *[gb|GU233535], micro.17361.c1) http://pgrc.ipk-gatersleben.de/poci or CAPS-based (*bch *[gb|GU233534]), and integrated using the same linkage mapping software. PCR primers and conditions and the restriction enzymes used for marker scoring, are listed in Additional file 4.

QTL analysis of quantitative data was performed using the software package MapQTL^® ^Version 5.0 [48]. The phenotypic data were tested for normality. QTL analysis was initially done using the interval mapping method [49]. Detection of a significant QTL was done using a genome-wide LOD threshold calculated with the permutation test option provided in the program. Gene expression data obtained with qRT-PCR were normalized against the reference gene and the relative expression levels were treated as a quantitative trait.

## Abbreviations

qRT-PCR: quantitative reverse transcriptase polymerase chainreaction; eQTL: expression Quantitative Trait Loci; TLRP: Tyrosine and Lysine-rich protein; Bch: β-carotene hydroxylase.

## Authors' contributions

BK performed the profiling experiments for the texture and flesh color traits, QTL analysis, mapping of candidate genes and wrote the manuscript together with CWBB. and RGFV. MO was responsible for all qRT-PCR work and analysis, as well as marker development. JU carried out and analyzed the methionine BSA expression profiling experiment. TA determined methionine content in the tubers and RDV determined carotenoids content and identification. All authors have read and approved the final manuscript.

## Supplementary Material

Additional file 1 **Supplementary table S1**. Candidate gene list for tuber flesh color trait. Click here for file

Additional file 2 **Supplementary table S2**. Candidate gene list for tuber cooking type trait. Click here for file

Additional file 3 **Supplementary table S3**. Candidate gene list for methionine content trait. Click here for file

Additional file 4 **Supplementary table S4**. Primers sequences for qRT-PCR and marker development. Click here for file
